# Testing of a Self-administered 6-Minute Walk Test Using Technology: Usability, Reliability and Validity Study

**DOI:** 10.2196/22818

**Published:** 2021-09-23

**Authors:** Jenna Smith-Turchyn, Scott C Adams, Catherine M Sabiston

**Affiliations:** 1 School of Rehabilitation Science McMaster University Hamilton, ON Canada; 2 Faculty of Kinesiology and Physical Education University of Toronto Toronto, ON Canada

**Keywords:** exercise, physical activity, usability testing, applications, mobile phone

## Abstract

**Background:**

The need to attend a medically supervised hospital- or clinic-based appointment is a well-recognized barrier to exercise participation. The development of reliable and accurate home-based functional tests has the potential to decrease the burden on the health care system while enabling support, information, and assessment.

**Objective:**

This study aims to explore the usability (ie, acceptability, satisfaction, accuracy, and practicality) of the EasyMeasure app to self-administer the 6-minute walk test (6MWT) in young, healthy adults and determine parallel form reliability and construct validity of conducting a self-administered 6MWT using technology.

**Methods:**

We used a usability study design. English-speaking, undergraduate university students who had access to an iPhone or iPad device running iOS 10 or later and self-reported ability to walk for 6 minutes were recruited for this study. Consenting participants were randomized to either a standard 6MWT group (ie, supervised without the use of the app) or a technology 6MWT group (ie, unsupervised with the app to mimic independent implementation of the test). All participants performed a maximal treadmill test. Participants in the 6MWT group completed the Unified Theory of Acceptance and Use of Technology (UTAUT) questionnaire and a satisfaction questionnaire after completing the assessment. Parallel form reliability of the 6MWT using technology was analyzed by comparing participant self-administered scores and assessor scores using Pearson correlation coefficients across and between trials. Construct validity was assessed by comparing participant 6MWT scores (both standard and using technology) with maximum treadmill test variables (peak oxygen uptake and ventilatory threshold [VT]).

**Results:**

In total, 20 university students consented to participate in the study. All but 2 participants (8/10, 80%) in the technology 6MWT group had deviations that prevented them from accurately conducting the 6MWT using the app, and none of the participants were able to successfully score the 6MWT. However, a significantly strong correlation was found (*r*=.834; *P*=.003) when comparing participants’ scores for the 6MWT using technology with the assessors’ scores. No significant correlations were found between maximal treadmill test peak oxygen uptake scores and 6MWT prediction equations using standard 6MWT scores (equation 1: *r*=0.119; *P*=.78; equation 2: *r*=0.095; *P*=.82; equation 3: *r*=0.119; *P*=.78); however, standard 6MWT scores were significantly correlated with VT values (*r*=0.810; *P*=.02). The calculated submaximal treadmill scores and assessor 6MWT scores using technology also demonstrated a significant correlation (*r*=0.661; *P*=.04).

**Conclusions:**

This study demonstrated significant usability concerns regarding the accuracy of a self-administered 6MWT using the EasyMeasure app. However, the strong and significant correlation between the 6MWT and VT values demonstrates the potential of the 6MWT to measure functional capacity for community-based exercise screening and patient monitoring.

## Introduction

### Background

Tests of mobility, physical functioning, and aerobic capacity are commonly used in research and clinical practice to evaluate the impact of exercise programs [1] and for prognostic prescreening and risk management purposes (eg, identifying individuals at risk for complications related to certain medical conditions and exercise participation) [2]. However, the need to attend a medically supervised hospital- or clinic-based screening assessment is a well-recognized barrier to exercise participation [2,3]. This barrier is likely heightened for individuals who are older, are living in rural and remote communities, are living with chronic conditions that limit their functional independence, and lack accessible health care services.

In Canada, there is a rapidly growing aging population wherein 1 in 4 adults live with 2 or more chronic conditions, and half of older adults live with three or more chronic conditions [4-6]. Furthermore, many individuals live in remote and rural communities, limiting health care availability. Therefore, it is becoming increasingly important to develop simple, self-administered, and home-based functional tests for community physicians and rehabilitation professionals to facilitate distance-based risk screening and pre-exercise clearances. The development of reliable and accurate home-based functional tests has the potential to decrease the burden on the health care system while enabling support, information, and assessments.

The 6-minute walk test (6MWT) is an easy to perform, submaximal, and widely used test of functional exercise capacity [7]. It is used clinically as an objective measure of functional status to determine appropriate exercise prescription and predict morbidity and mortality [7]. It measures the distance covered in 6 minutes, with the objective being to walk as far as possible at a comfortable pace within those 6 minutes [7]. The 6MWT has been used in people across the lifespan (eg, aged 2-65+ years) and a range of health conditions (eg, stroke, pulmonary diseases, osteoarthritis, and dementia) [7], with established age- and condition-specific normative data available by sex [1]. The 6MWT has demonstrated responsiveness to assess change in functional exercise capacity, and minimal clinically important differences for various populations, ranging from 19 to 49 m, have been established [8,9]. In addition, the ability to walk approximately 288 to 300 m in 6 minutes has been suggested as a threshold for functional independence and community ambulation [10,11]. As the 6MWT is widely used clinically as a test of functional exercise capacity, it is important to validate its ability to estimate peak oxygen uptake (VO_2peak_) to ensure its outcomes are being used safely and reliably. A cardiopulmonary exercise test (CPET) is the gold standard for assessing VO_2peak_. However, CPET requires specialized equipment and personnel that are not widely available, particularly in rural and remote communities. Currently, correlation coefficients for the 6MWT and VO_2peak_ reported in the literature range in value [12,13]. Given this evidence and the common clinical use of the 6MWT, it is important for researchers to continue to explore the accuracy of estimating VO_2peak_ from 6MWT data using a variety of predictive equations.

The EasyMeasure app [14] shows the distance from the phone to an object, as seen through the lens of the iPhone or iPad camera. It is free to download on any Apple iPhone, iPad, or iPod device that has iOS 10.0 or later installed. This app can be useful in conducting a self-administered 6MWT by allowing users to measure the distance to an object before beginning the walking test. This could aid in calculating the total distance walked at the end of 6 minutes. The EasyMeasure app does not include a lap counter or timer as part of its functions. To date, no study has assessed the use of the EasyMeasure app as a tool to self-administer the 6MWT in any population.

### Objective

The primary objective of this study is to explore the usability (ie, acceptability, satisfaction, accuracy, and practicality) of the EasyMeasure app to self-administer the 6MWT in young, healthy adults. Our secondary objectives are to determine the parallel form reliability and construct validity of conducting a self-administered 6MWT using technology. The results of this trial in a healthy young adult population will help determine the updates and changes necessary for successful implementation before use with other populations.

## Methods

### Study Design

A usability study design was used to test the app characteristics, parallel form reliability, and construct validity of conducting a self-administered 6MWT using technology in a controlled setting. Participants were asked to perform either a self-administered 6MWT using the EasyMeasure app or a traditional investigator-supervised 6MWT in the laboratory. All participants were also asked to perform a maximal treadmill test for aerobic capacity. The University of Toronto Research Ethics Board approved this study (#37108).

### Participants and Recruitment

We recruited 20 undergraduate university students via email within the Faculty of Kinesiology and Physical Education and among varsity athletes from the University of Toronto. Eligible participants included (1) English-speaking (2) undergraduate university students (3) younger than 30 years (4) who had access to an iPhone or iPad device running iOS 10 or later, (5) were willing to download the EasyMeasure app on their device, and (7) self-reported being able to walk for 6 minutes. Potential participants were excluded from the study if they (1) self-reported having any physical injury or condition that precluded them from walking safely for 6 minutes or (2) self-reported a cognitive condition that precluded them from understanding instructions or the consent form provided. Interested respondents contacted the study investigators to schedule an assessment session date and time. All participants were required to complete written informed consent before beginning the project.

### Procedure

#### Preparation

Eligible consenting participants were randomized to either the standard 6MWT group (ie, supervised without the app) or the technology 6MWT group (ie, unsupervised with the EasyMeasure app to mimic independent implementation of the test). Before the testing session, all participants were asked to download the EasyMeasure app onto their devices. Upon arrival at the testing sessions, participants were informed of which version of the 6MWT they would complete. Participants’ heart rate, blood pressure, rate of perceived exertion (RPE), and oxygen saturation levels were assessed before and after the testing sessions to ensure participant safety.

#### Standard 6MWT

Participants in the standard group were given the 6MWT instructions by an assessor (physiotherapist [JST] or exercise physiologist [SCA]). Participants were asked to walk as quickly as possible in a comfortable manner for 6 minutes along a previously measured straight pathway. During the test, participants were timed by the assessor and given standard encouragement at each minute interval. The assessor counted the number of laps performed by each participant. At 6 minutes, participants stopped at their location along the path, and the assessor measured the total distance walked for the final lap. The assessor calculated the total distance walked in 6 minutes and interpreted the participants’ test scores.

#### 6MWT Using Technology

Compared with the standard 6MWT group, participants in the technology group had to measure the distance between objectives (measure the test path), time the test, and count laps independently. To accomplish this, participants in the technology group were given instructions by the assessor on how to use the EasyMeasure app (including instructions for proper calibration of the app as well as how to measure the distance to an object and how to take a photo of the distance recorded), how to perform the 6MWT, and how to interpret their 6MWT scores. Participants used the app to measure the distance from the starting point to a predefined object. They recorded the distance between the starting point and the object by taking a still image using the app. Participants then walked consistently for 6 minutes around the 2 objects. Independent of the EasyMeasure app, they timed themselves using their cell phones and counted laps (either within their head or with the counter function on their phone). At the completion of the 6 minutes, they used the EasyMeausre app to measure the distance walked along the path during their final lap. They recorded this distance by taking a still image using the app. After performing the test, participants calculated the results of their test (ie, how many meters they walked in 6 minutes) by multiplying the number of laps walked by the distance measured in the app. They then interpreted their test scores by comparing their 6MWT score with provided normative values for age and sex (ie, determine if their scores were within normal limits for their age range and state if they were safe to exercise independently based on results).

This test was performed autonomously but in the laboratory. An assessor was present but did not interfere with or provide encouragement. The assessor knew the distance from the starting line to the object of measurement and counted the laps the participants completed to track accuracy; however, participants were not aware of the assessor’s count. The assessor also made notes on the number of deviations to instructions made by participants, the ability of participants to successfully report and interpret their scores, and if any additional resources were needed by participants.

After completing the test, participants in this group completed the Unified Theory of Acceptance and Use of Technology (UTAUT) questionnaire and a satisfaction questionnaire. The UTAUT is an 18-item self-report measure used to explain individuals’ intentions to use a form of technology. It holds four key constructs, including (1) performance expectancy (the extent to which the individual believes that use of the technology will lead to improved health), (2) effort expectancy (how easy was the use of technology perceived to be), (3) social influence (the extent to which an individual believes that others want them to use this technology system), and (4) facilitating conditions (to what extent did an individual believe there is the organizational and technical infrastructure to support the use of this process) [15]. Each item was graded on a 5-point Likert scale ranging from 1 (strongly disagree) to 5 (strongly agree) [15]. The satisfaction questionnaire allowed individuals to describe the positive and negative aspects of using this approach to conduct a 6MWT and their thoughts on the practicality of performing these tests in this manner alone at home. The survey had 8 questions that were measured on a 7-point Likert scale from 1 (not at all) to 7 (extremely) and two open-ended questions at the end where participants gave additional details as to what they liked and did not like about using the app to perform the 6MWT. This survey was pilot-tested by a study investigator in a previous project [16].

#### Maximal Treadmill Test

Following the 6MWTs, VO_2peak_ was assessed via a CPET on a treadmill under the supervision of a certified exercise physiologist (SCA) using an individualized protocol [17]. Briefly, participants began by performing a 5-minute warm-up at a 0% incline at a belt speed sufficient to elicit approximately 60% of their age-predicted maximal heart rate. The test continued using the constant individualized belt speed established during the warm-up, with the incline increasing by 2% every 2 minutes until exhaustion. Participants’ oxygen uptake (TrueOne 2400, Parvo Medics) and heart rate (FT4 HR monitor, Polar) were measured continuously. Blood pressure and RPE were recorded every 2-4 minutes. VO_2peak_ was defined as the highest 15-second average value for oxygen uptake recorded during the test. The maximal effort was defined as participants achieving at least two of the following criteria: (1) leveling off of oxygen uptake despite an increase in workload, (2) respiratory exchange ratio >1.1, and (3) RPE ≥9/10 [18]. The ventilatory threshold (VT) was estimated using the V-slope method [19].

### Sample Size

The sample size for this study was determined based on informal guidelines for usability (ie, acceptability, satisfaction, accuracy, and practicality), suggesting a group size of 3-20 participants [20]. By the end of the trial, we ensured that no new problems arose during subject performance (saturation of data) to ensure we had included enough participants to address the main study aim.

### Data Analysis

Quantitative data were summarized using descriptive statistics (ie, means and SDs reported for continuous data; frequencies and percentages reported for categorical data). The open-ended survey questions were analyzed using qualitative descriptive analysis, and responses were grouped into meaningful categories that arose from the data. Parallel form reliability of the 6MWT using technology was analyzed by comparing participant self-administered scores with assessor scores using Pearson correlation coefficients across and between trials. A *t* test was used to determine the statistical significance between the assessor and participant scores. Construct validity was assessed by comparing participant 6MWT scores (both standard and using technology) with CPET-derived variables (ie, VO_2peak_ and VT). As there is no standardized way to convert 6MWT values to VO_2peak_ estimates, 3 commonly used predictive equations were used to estimate VO_2peak_ from 6MWT scores for each participant. Using 3 different equations, as opposed to choosing one, allowed consideration of a larger scope of possible VO_2peak_ values when comparing outcomes. The estimated VO_2peak_ values for each equation were then plotted on a scatterplot to identify the outliers. The remaining scores were then correlated to actual VO_2peak_ values obtained from the maximal treadmill test using Spearman correlation coefficients across and between trials to determine the strength of the relationship. The correlations between the 6MWT and VT values were similarly assessed using Spearman correlation coefficients to examine the ordinal relationship between the 2 variables. All statistical analyses were conducted using STATA (version 15, StataCorp) with the significance set at *P*<.05.

## Results

### Participant Characteristics

In total, 20 university students consented to participate in this study; 10 participants were randomized to each group. Most participants (16/20, 80%) were female with a mean age of 20.1 (SD 2.2) years. Participants had a mean height of 165.8 (SD 7.8) cm and a mean weight of 65.0 (SD 10.9) kg. Participant characteristics and VO_2peak_ estimates for each group are shown in Table 1. Overall, participants in the standard 6MWT group walked significantly further during the test than those in the self-administered 6MWT group (mean difference 163.4, 95% CI 95.4-231.5; *P*=.001).

**Table 1:** Participant characteristics and 6MWT^a^ results.

Characteristic	Standard 6MWT group (n=10)	6MWT using technology (n=10)
Age (years), mean (SD)	20.6 (2.91)	19.5 (1.18)
Sex (female), n (%)	8 (80)	8 (80)
Height (cm), mean (SD)	168.22 (6.87)	163.44 (8.22)
Weight (kg), mean (SD)	65.55 (11.17)	65.52 (11.36)
6MWT distance (m), mean (SD)	658.74 (62.69)	495.30 (80.95)^b^
VO_2peak_^c^ estimate 1^d^, mean (SD)	23.74 (1.88)	19.56 (4.75)
VO_2peak_ estimate 2^e^, mean (SD)	24.45 (1.62)	21.41 (3.78)
VO_2peak_ estimate 3^f^, mean (SD)	20.10 (1.44)	16.90 (3.64)

^a^6MWT: 6-minute walk test.

^b^Assessor score.

^c^VO_2peak_: peak oxygen uptake.

^d^Peak oxygen uptake = 0.03 × distance (m) + 3.98 [21].

^e^Peak oxygen uptake = 0.02 × distance (m) − 0.191 × age (years) − 0.07 × weight (kg) + 0.09 × height (cm) + 0.26 × (rate pressure product × 10^−3^) + 2.45 [21].

^f^Peak oxygen uptake = 4.948 + 0.023 × distance (m) [22].

### Usability Outcomes

A total of 10 participants completed the 6MWT using this technology. All but 2 participants (8/10, 80%) had deviations that prevented them from accurately conducting the test using the EasyMeasure app. This included 60% (6/10) of participants who lost the count of laps, 40% (4/10) who did not walk at their maximum pace, and 10% (1/10) who did not measure the distance of their last lap.

None of the participants were able to successfully score (ie, calculate the actual distance covered in 6 minutes) the 6MWT; 60% (6/10) of participants did not count the number of laps correctly, and 60% (6/10) measured the distance of each lap incorrectly by ≥0.5 m. In total, 30% (3/10) of participants interpreted their scores incorrectly, reporting that they were within normal limits for their age and sex when they were not; 40% (4/10) of participants identified that they required additional resources to conduct the test successfully, with 20% (2/10) of participants suggesting the need for a lap counter and a calculator.

### UTAUT Questionnaire

Table 2 summarizes the participants’ responses to the UTAUT questionnaire. All effort expectancy question scores had a median value of 4 (agree) or better, demonstrating that participants found the process of conducting the 6MWT using the EasyMeasure app easy to perform. The median scores for all facilitating condition questions were high (at 5, strongly agree) and low for technology anxiety questions (2 or less, disagree), indicating that participants felt they had appropriate knowledge and skill to comfortably use the EasyMeasure app to perform the 6MWT. When asked if they would be willing to use a system such as this in their health care, most participants indicated that they would (demonstrated by a median score of 4, agree).

**Table 2:** Unified Theory of Acceptance and Use of Technology results.

Question		1 (strongly disagree), n (%)	2 (disagree), n (%)	3 (neither disagree or agree), n (%)	4 (agree), n (%)	5 (strongly agree), n (%)	Mean score (SD)	
**PE^a^** **(out of 15)**								10.3 (1.25)
	PE1	0 (0)	1 (10)	6 (60)	3 (30)	0 (0)	3.2 (0.63)	
	PE2	0 (0)	0 (0)	2 (20)	7 (70)	1 (10)	3.9 (0.57)	
	PE3	0 (0)	1 (10)	6 (60)	3 (30)	0 (0)	3.2 (0.63)	
**EE^b^** **(out of 15)**								12.8 (1.75)
	EE1	0 (0)	0 (0)	1 (10)	6 (60)	3 (30)	4.2 (0.63)	
	EE2	0 (0)	0 (0)	1 (10)	4 (40)	5 (50)	4.4 (0.70)	
	EE3	0 (0)	0 (0)	1 (10)	6 (60)	3 (30)	4.2 (0.63)	
**SI^c^** **(out of 15)**								9.60 (2.01)
	SI1	1 (10)	3 (30)	5 (50)	1 (10)	0 (0)	2.6 (0.84)	
	SI2	0 (0)	0 (0)	4 (40)	5 (50)	1 (10)	3.7 (0.67)	
	SI3	1 (10)	2 (20)	2 (20)	3 (30)	2 (20)	3.3 (1.34)	
**FC^d^** **(out of 20)**								18.9 (2.60)
	FC1	0 (0)	0 (0)	1 (10)	1 (10)	8 (80)	4.7 (0.67)	
	FC2	0 (0)	0 (0)	1 (10)	1 (10)	8 (80)	4.8 (0.79)	
	FC3	0 (0)	0 (0)	1 (10)	0 (0)	9 (90)	4.8 (0.63)	
	FC4	0 (0)	1 (10)	0 (0)	1 (10)	8 (80)	4.6 (0.97)	
**ANX^e^** **(out of 15)^f^**								4.3 (1.15)
	ANX1	7 (70)	3 (30)	0 (0)	0 (0)	0 (0)	1.3 (0.48)	
	ANX2	4 (40)	5 (50)	1 (10)	0 (0)	0 (0)	1.56 (0.70)	
	ANX3	5 (50)	4 (40)	1 (10)	0 (0)	0 (0)	1.6 (0.70)	
**B^g^ (out of 10)**								5.9 (2.33)
	B2	2 (20)	3 (30)	2 (20)	2 (20)	1 (10)	2.7 (1.33)	
	B3	1 (10)	2 (20)	1 (10)	6 (60)	0 (0)	3.2 (1.14)	

^a^PE: performance expectancy.

^b^EE: effort expectancy.

^c^SI: social influence.

^d^FC: facilitating conditions.

^e^ANX: technology anxiety.

^f^Performance expectancy, effort expectancy, social influence, facilitating conditions, behavioral intention to use scales: a higher score is better (eg, higher performance expectancy); for the technology anxiety scale, a lower score is better (lower anxiety).

^g^B: behavioral intention to use.

### Satisfaction Questionnaire

Table 3 summarizes the participant responses for each question of the satisfaction questionnaire. When asked about the positive aspects of the 6MWT using technology, 60% (6/10) of participants appreciated that it was easy to use and set up, 50% (5/10) liked that it was accessible and free for everyone, 30% (3/10) appreciated the accuracy of measurement, 10% (1/10) liked that it could be used at home, and 10% (1/10) liked that it was quick to perform. Negative aspects reported by participants included that the app did not provide information directly related to the 6MWT (reported by 5/10, 50% of participants). Specifically, the app did not count the number of laps completed, and they had to calculate the total distance walked on their own. Moreover, 20% (2/10) of participants did not like that the distance between the 2 objects was small. Other negative aspects included that the app had distracting advertisements (1/10, 10%) and required a smartphone (1/10, 10%). Only 10% (1/10) of participants questioned the accuracy of the app’s ability to measure distance.

**Table 3:** Satisfaction questionnaire results.

Question	Median score^a^
1. How logical does the use of the EasyMeasure app to conduct a self-administered 6MWT^b^ seem to you?	5.5
2. How scientific does this way of testing the 6MWT seem to you?	5
3. How complete does this way of testing the 6MWT seem to you? In other words, do you think this method covers all of the necessary steps of performing this test to get an accurate value? Would you need any other resources?	5
4. To what extent would this form of self-evaluation help an individual assess their performance capacity?	5
5. How likely would you be to use this method to assess your 6MWT score if it was available to you?	4.5
6. How likely would you be to assess your 6MWT score in this capacity at home, compared to having a certified health care professional perform this test for you at another location?	5.5
7. How effective do you think this method to assess a 6MWT score would be for most people?	4
8. If a close friend or relative wanted to assess their walking capacity, would you recommend they use this method to test?	5

^a^Scored from 1 (not at all) to 7 (extremely).

^b^6MWT: 6-minute walk test.

### Reliability

**Figure 1:**
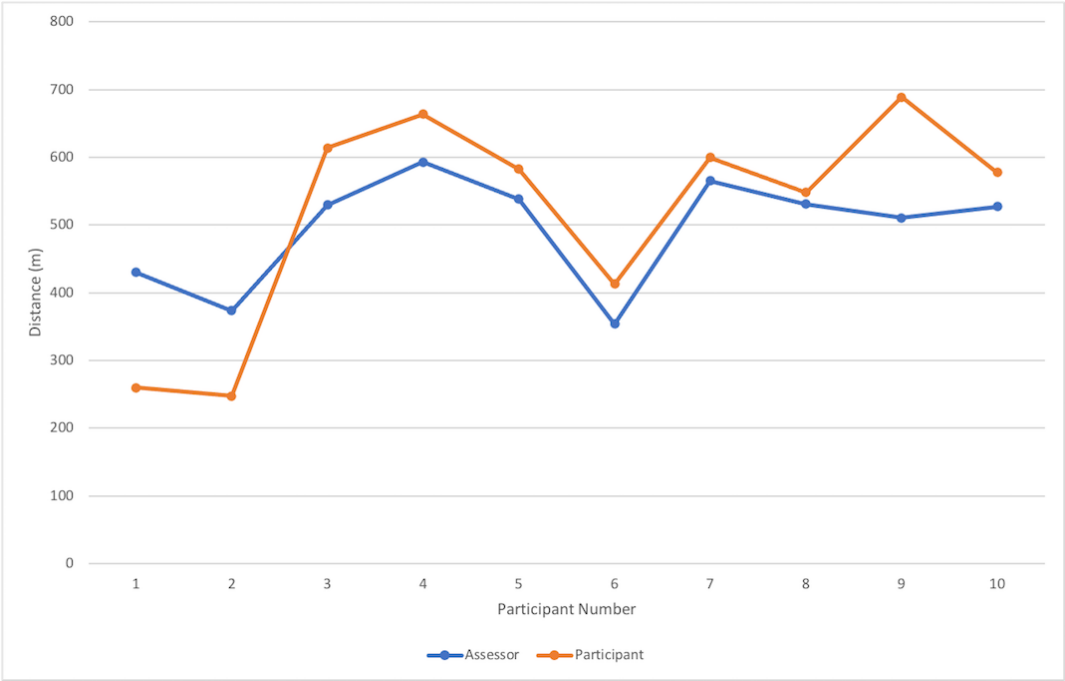
Participant versus assessor scores using 6-minute walk test technology.

A significantly strong correlation was found (*r*=0.834; *P*=.003) when comparing participants’ scores (self-determined total distance walked) for the 6MWT using technology with the assessors’ scores (actual distance walked). No statistically significant difference was found between the participant and assessor scores (*t*_9_=0.4319; *P*=.67). However, when comparing differences between participant and assessor scores, all values were greater than the 6MWT mean clinically important difference (MCID) values, demonstrating inaccuracy between the 2 measures. Figure 1 shows a comparison of the assessor and participant scores.

### Validity

#### Standard 6MWT

After reviewing the outcomes on a scatterplot, two participant scores were removed as outliers. The remaining scores demonstrated no significant correlation between maximal treadmill test VO_2peak_ scores and any of the 6MWT prediction equations using the standard 6MWT scores (equation 1: *r*=0.119; *P*=.78; equation 2: *r*=0.095; *P*=.82; equation 3: *r*=0.119; *P*=.78). However, the 6MWT scores were significantly correlated with VT values (*r*=0.810; *P*=.01).

#### 6MWT Using Technology

Owing to inaccuracy in participant scores when performing the self-administered 6MWT using technology, comparisons were only made between assessor scores and maximal treadmill test VO_2peak_ scores. After reviewing the scores on a scatterplot, no outliers were removed. A significant correlation was found between equation 2 and the 6MWT scores (equation 2: *r*=0.721; *P*=.02). No significant correlations were demonstrated for the 2 other equations (equation 1: *r*=0.576; *P*=.08; equation 3: *r*=0.576; *P*=.08), although it is acknowledged that the correlation coefficients are at least moderate in strength and may suggest meaningful associations. The calculated submaximal treadmill scores and assessor 6MWT scores using technology demonstrated a significant correlation (*r*=0.661; *P*=.04).

#### Assessor Feedback and Learnings

After observing the participants in the 6MWT group, the assessor noted commonalities in participant behavior. First, many young adults in this group were not perceived to be walking at their maximum speed, as instructed by the assessor. It appeared to be difficult for this group to multitask (walk, count laps, and time themselves) and correctly interpret their 6MWT scores. For example, even when scores obtained were below normal age-matched values (most often due to not walking at maximum walking speed), they often said they were *safe to exercise* based on their perception of their overall health. Together, these observations may help to explain the inaccurate findings of participants in the technology 6MWT group when compared with those in the standard 6MWT group.

## Discussion

### Principal Findings

The usability, reliability, and validity of conducting a self-administered 6MWT using a distance measurement app was explored among healthy young adults. The results of this study suggest that participants accepted the EasyMeasure app to perform the 6MWT. However, a primary finding of this study is that participants were unable to accurately self-administer and interpret the results of the 6MWT using this app. This finding suggests that the autonomously implemented 6MWT may not be feasible. Overall, these findings suggest a need to update the app and develop a more accurate process for measuring and interpreting the 6MWT before it can be used for clinical and research purposes. Our findings are particularly concerning given that younger university students are adept at using technology and applying simple standards for interpreting their results compared with older individuals living with or without chronic comorbidities [23].

Interestingly, our findings of inaccuracy are not consistent with those from a related study that tested an investigator-developed 6MWT app in older patients with chronic heart failure and hypertension [24]. The authors found that participants accurately and reliably measured the distance covered during the 6MWT using an app both within the laboratory and at home [24]. Participants in both studies reported that the apps were simple and easy to use independently [24]; however, the methods of measurement and app characteristics differed between the two studies and likely contributed to some of the inaccuracy observed in our study. Specifically, in the study by Brooks et al [24], participants were not required to count the number of laps walked or the distance between the starting point and endpoint of a single lap; the app did this for them and minimized the number of potential sources of measurement error. An advantage of the EasyMeasure app is that it is freely available to the public for download. However, the observed inaccuracy associated with our protocol using this technology solution negates the benefits of its accessibility. Furthermore, the age of the study participants was different. Older adults have less experience using technology and higher levels of technology-related anxiety than younger adults [23,25]. However, there is more research surrounding the needs of these individuals to successfully use technology in research and health care [23,25,26]. The younger participants in this study appeared to be quite comfortable using technology; however, this increased comfort with technology may have led to a decreased attention to the technology-related instructions provided and to the use of the various app settings in general [27].

The parallel form reliability findings revealed a significantly strong correlation between participants’ self-administered 6MWT scores and assessor scores for the group using technology. However, the differences between participant and assessor scores all exceeded the MCID values for the 6MWT (ie, 19-49 m [10,11]). MCID is defined as the smallest difference in a score on an outcome where patients perceive a benefit and hence mandates a change in the patient’s management [28]. The MCID was introduced to ensure that the outcomes of clinical trials were meaningful for the patient. In many instances, statistical significance is necessary but not sufficient [29]. As 6MWT MCID values were reached when comparing the differences between participant and assessor scores in all instances, concern arose as these differences could be interpreted as a meaningful difference to patients and affect the treatment they receive. Therefore, this result should be interpreted with caution.

Finally, validity results from this project found that 6MWT scores were significantly and strongly correlated with maximal treadmill test VT scores. This finding demonstrates that the 6MWT may be a valid measure of functional capacity and a marker of functional independence for clinicians to use when screening and monitoring patients in community settings. However, the results of this study showed that the 6MWT scores did not correlate with the maximal treadmill test VO_2peak_ scores. In this study, the 6MWT consistently underestimated VO_2peak_. There is variability in the literature regarding this outcome, with some studies demonstrating the validity of the 6MWT in predicting VO_2peak_ [30,31] and others demonstrating that the 6MWT is not a valid test to predict VO_2_peak [32]. This inconsistency is likely because the 2 tests measure different functional capabilities, and although the 6MWT may require near-maximal effort in some frail or impaired populations, it is not a valid measure of maximal oxygen uptake in many populations. Owing to the variability in correlation outcomes, it may be worthwhile to explore the use of other tests that could be self-administered to use as a predictor of functional capacity. For example, the Siconolfi step test [33] is a test in which participants are required to step up and down from a 10-inch step for a maximum of three 3-minute stages with increasing step rates [33]. It is a test that can be performed in any setting and is validated to predict VO_2peak_ in healthy adults [34,35] and those with a variety of chronic conditions [33,36,37]. Future studies should look at the potential of having this test be self-administered and compare different formats of functional capacity tests to determine which is most accurate and which participants are most satisfied with. Furthermore, more research is needed to test the effects of autonomously implemented functional capacity tests in older adults. A systematic review examining the use of mobile phones for health in older adults found 21 studies using distance-based interventions, and none of the programs included functional capacity assessment [38]. The concern is that interventions are delivered without appropriate baseline assessments or clearance.

### Future Research

On the basis of the findings of this study, it is recommended that the app used to self-administer the 6MWT be redesigned. Future apps should include functions that count laps, measure total distance walked, and time the test for users. This would help to overcome participant errors demonstrated in this study because of difficulty counting test laps and miscalculation of the total distance walked. In addition, several modifications to our tested methods should be considered to help overcome the usability issues identified in this study. The primary recommendation is to provide more detailed information and education to participants regarding the methods needed to accurately perform the test. This should include training videos or written instructions in addition to verbal instructions on how to calibrate the app to accurately measure the distance walked and information on how to perform and score the test. This would allow participants to review instructions before beginning the test, which may be most important if the test is being used with older adults or individuals who report a lack of competence with new technologies [38,39]. Other recommendations include allowing participants to have a *training run* before fully scoring the test and obtaining verbal feedback on performance for the first test, which could be completed virtually by a health care professional trained in scoring the test. The verbal feedback and encouragement given to the participants in the standard 6MWT group may have motivated them to walk faster and achieve a higher 6MWT score compared with participants in the technology group who did not have the same encouragement [40]. A *training run* may also serve to provide motivation and encouragement in the future.

### Limitations

The results of this study should be viewed with an understanding of their limitations. Testing of the self-administered 6MWT, which was designed to mimic a home-based test, took place in a laboratory setting. Although these tests were implemented in a room that was roughly the size of a large living room, we recognize that this does not reflect the space available to many people and suggest adding a third home-based arm in future studies. Adding a third home-based arm would be ideal because it would allow researchers to differentiate between issues resulting from measurement tools and measurement settings. In addition, the small sample size was determined based on usability study recommendations, and a larger sample with more diverse characteristics should be used for future testing and power considerations. A limitation of the 6MWT is evidence of a ceiling effect [41]; therefore, it is thought to be a more useful measure in older, deconditioned individuals than in young able-bodied populations.

### Conclusions

In conclusion, this study demonstrated significant usability concerns regarding the accuracy of a self-administered 6MWT using the EasyMeasure app. Despite the reported ease of use of this technology, the inaccurate measurements and challenges associated with interpreting the test scores suggest that the app design and tested protocol are of limited use for research and clinical purposes. However, the strong and significant correlation between the 6MWT and VT values demonstrates the potential of the 6MWT to measure functional capacity for community-based exercise screening and patient monitoring. Further research is needed to develop a more accurate means of implementing and interpreting a self-administered 6MWT to facilitate pre-exercise screening and patient assessment for distance-based health care and research purposes.

## Abbreviations

6MWT: 6-minute walk test
CPET: cardiopulmonary exercise test
MCID: mean clinically important difference
RPE: rate of perceived exertion
UTAUT: Unified Theory of Acceptance and Use of Technology
VO2peak: peak oxygen uptake
VT: ventilatory threshold
